# Identification and Comparative Expression Analysis of Interleukin 2/15 Receptor β Chain in Chickens Infected with *E. tenella*

**DOI:** 10.1371/journal.pone.0037704

**Published:** 2012-05-25

**Authors:** Jipseol Jeong, Woo H. Kim, Jeongmi Yoo, Changhwan Lee, Suk Kim, Jae-Hyeon Cho, Hyung-Kwan Jang, Dong W. Kim, Hyun S. Lillehoj, Wongi Min

**Affiliations:** 1 College of Veterinary Medicine and Research Institute of Life Science, Gyeongsang National University, Jinju, Korea; 2 Departments of Infectious Diseases and Avian Diseases, College of Veterinary Medicine and Korea Zoonosis Research Institute, Chonbuk National University, Jeonju, Korea; 3 National Institute of Animal Science, RDA, Cheonan, Chungnam, Korea; 4 Animal Parasitic Diseases Laboratory, Animal and Natural Resources Institute, Agricultural Research Service, United States Department of Agriculture, Beltsville, Maryland, United States of America; Institut national de la santé et de la recherche médicale (INSERM), France

## Abstract

**Background:**

Interleukin (IL) 2 and IL15 receptor β chain (IL2/15Rβ, CD122) play critical roles in signal transduction for the biological activities of IL2 and IL15. Increased knowledge of non-mammalian IL2/15Rβ will enhance the understanding of IL2 and IL15 functions.

**Methology/Principal Findings:**

Chicken IL2/15Rβ (chIL2/15Rβ) cDNA was cloned using 5′/3′-RACE. The predicted protein sequence contained 576 amino acids and typical features of the type-I cytokine receptor family. COS-7 cells transfected with chIL2/15Rβ produced proteins of approximately 75 and 62.5 kDa under normal and tunicamycin-treated conditions, respectively. The genomic structure of chIL2/15Rβ was similar to its mammalian counterparts. chIL2/15Rβ transcripts were detected in the lymphoblast cell line CU205 and in normal lymphoid organs and at moderate levels in bursa samples. Expression profiles of chIL2/15Rβ and its related cytokines and receptors were examined in ConA-stimulated splenic lymphocytes and in ceca-tonsils of *Eimeria tenella*-infected chickens using quantitative real-time PCR. Expression levels of chIL2/15Rβ, chIL2Rα, and chIL15Rα were generally elevated in ceca-tonsils and ConA-activated splenic lymphocytes. However, chIL2 and chIL15 expression levels were differentially regulated between the samples. chIL2 expression was upregulated in ConA-activated splenic lymphocytes, but not in ceca-tonsils. In constrast, chIL15 expression was upregulated in ceca-tonsils, but not in ConA-activated splenic lymphocytes.

**Conclusions/Significance:**

We identified an avian form of IL2/15Rβ and compared its gene expression pattern with those of chIL2, chIL15, chIL2Rα, and chIL15Rα. Our observations suggest that chIL15 and its receptors, including chIL2/15Rβ, play important roles in mucosal immunity to intestinal intracellular parasites such as *Eimeria*.

## Introduction

Interleukin 2 (IL2) and IL15 play key roles in the regulation of homeostasis and function of T cells and natural killer (NK) cells. The effects of these cytokines on target cells are mediated by their heterotrimeric receptors that consist of a specific α-subunit and two shared subunits, IL2 and IL15 receptor β (IL2/15Rβ, CD122) and a common cytokine receptor γ (γ_c_) [1], [2]. Although the α-subunits of the IL2 and IL15 receptors define the binding specificity of the cytokines, the subunits do not participate directly in intracellular signaling due to their short cytoplasmic tails. Thus, the cytoplasmic regions of IL2/15Rβ and γ_c_, members of the type-I cytokine receptor family that display a characteristic spacing of four conserved cysteine residues and a WSXWS motif, bind to intracellular signaling molecules and transmit intracellular signals [3]–[5].

The gene encoding IL2/15Rβ is composed of ten exons and nine introns, resulting in an 80 kDa transmembrane receptor. IL2/15Rβ is expressed on NK cells and CD8^+^ T cells and is present on activated CD4^+^ T cells, B cells, and monocytes [6]–[8]. IL2/15Rβ plays critical functions in the regulation of lymphoid development, differentiation, and homeostasis in both innate and adaptive immunity. Expression of IL2/15Rβ has been identified in normal, non-immune tissues, such as neurons and glial cells of adult rat brains, suggesting involvement in nerve regeneration [9]. Furthermore, soluble or truncated IL2/15Rβ forms have been detected in sera from healthy individuals and patients with inflammatory bowel disease and in lymphoid cell lines [10]–[12]. IL2/15Rβ-related disorders have been reported in which defective IL2/15Rβ expression leads to abnormal development of NK cells and intestinal intraepithelial lymphocytes [13], [14] and autoimmunity [15]–[17]. Blockade of IL-2/15Rβ results in splenic NK cell deficiency [18] and effectively resolves autoimmune intestinal damage induced by elevated levels of IL15 in IL15-transgenic mice [19].

The avian immune system provides an important model for the study of basic and applied immunology. Despite the general lack of cross-reactivity and the low level of sequence conservation between avian and mammalian cytokines, many chicken genes are homologous to their mammalian counterparts, including IL2 [20], IL15 [21], IL2 receptor α chain (IL2Rα) [22], and IL15Rα [23]. Avian species, unlike mammals, express two different γ_c_ transcripts due to alternative splicing [24], [25]. Little information, however, is available on chicken IL2/15Rβ (chIL2/15Rβ). Therefore, we examined full-length cDNA and the genomic structure encoding a chicken homologue of mammalian IL2/15Rβ. Through quantitative real-time PCR and Western blot analyses, tissue distribution of chIL2/15Rβ transcripts and molecular weights were analyzed. Furthermore, quantitative real-time PCR was used to evaluate expression profiles of chIL2/15Rβ and related cytokines and receptors in ConA-stimulated splenic lymphocytes and ceca-tonsils and spleens from chickens infected with *E. tenella*.

## Results

### Cloning and Characterization of chIL2/15Rβ cDNA

**Figure 1:**
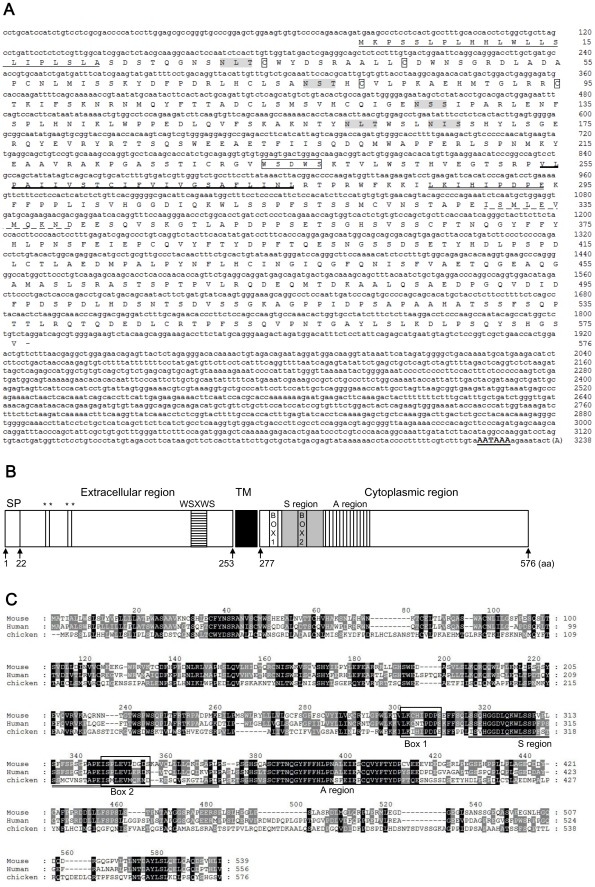
Molecular features of the chicken IL2/15Rβ cDNA. (A) Nucleotide sequence and predicted amino acid sequence of chicken IL2/15Rβ. The predicted signal peptide is single-underlined. The conserved cysteine residues and WSXWS motif are boxed. Five potential N-linked glycosylation sites in the extracellular region are highlighted. The putative transmembrane region is indicated by the bold underline. Box 1 and Box 2 domains are indicated by double underline and dashed underline, respectively. The polyadenylation signal is in bold and underlined. (B) Schematic representation of chIL2/15Rβ cDNA. SP, predicted signal peptide; *, conserved cysteine residues; WSXWS, WSXWS motif; TM, putative transmembrane region; S region, serine-rich region; A region, acidic region. (C) Multiple alignment of amino acid sequences of chIL2/15Rβ with mammalian homologues. The multiple alignment was generated with ClustalW2 and the conserved amino acid residues in these sequences are shaded to show homology. Box 1 and Box 2 domains are boxed. A and S regions are indicated by single and double underlines, respectively.

The full-length cDNA of chIL2/15Rβ was cloned using 5'/3'-RACE based on the sequence information of an EST fragment (accession No. ENSGALG00000012472). The chIL2/15Rβ cDNA was approximately 3.2 kb and contained a 1731 bp open reading frame (ORF) predicted to encode a putative 576 amino acid protein with a molecular weight of 61.4 kDa (non-glycosylated) and a predicted isoelectric point of 5.15. The predicted chIL2/15Rβ amino acid sequence contained a leader sequence (amino acids 1–22), an extracellular domain (amino acids 23–253), a transmembrane domain (amino acids 254–276), and a cytoplasmic domain (amino acids 277–576) (Fig. 1A). Amino acid sequence comparison using ClustalW (www.ebi.ac.uk/Tools/clustalw2) indicated that chIL2/15Rβ shared 27–29% identity with its human, mouse, and rat counterparts [26]–[28], and 18–22% identity with those from zebrafish, salmon, and rainbow trout [29] (Table 1).

**Figure 2:**
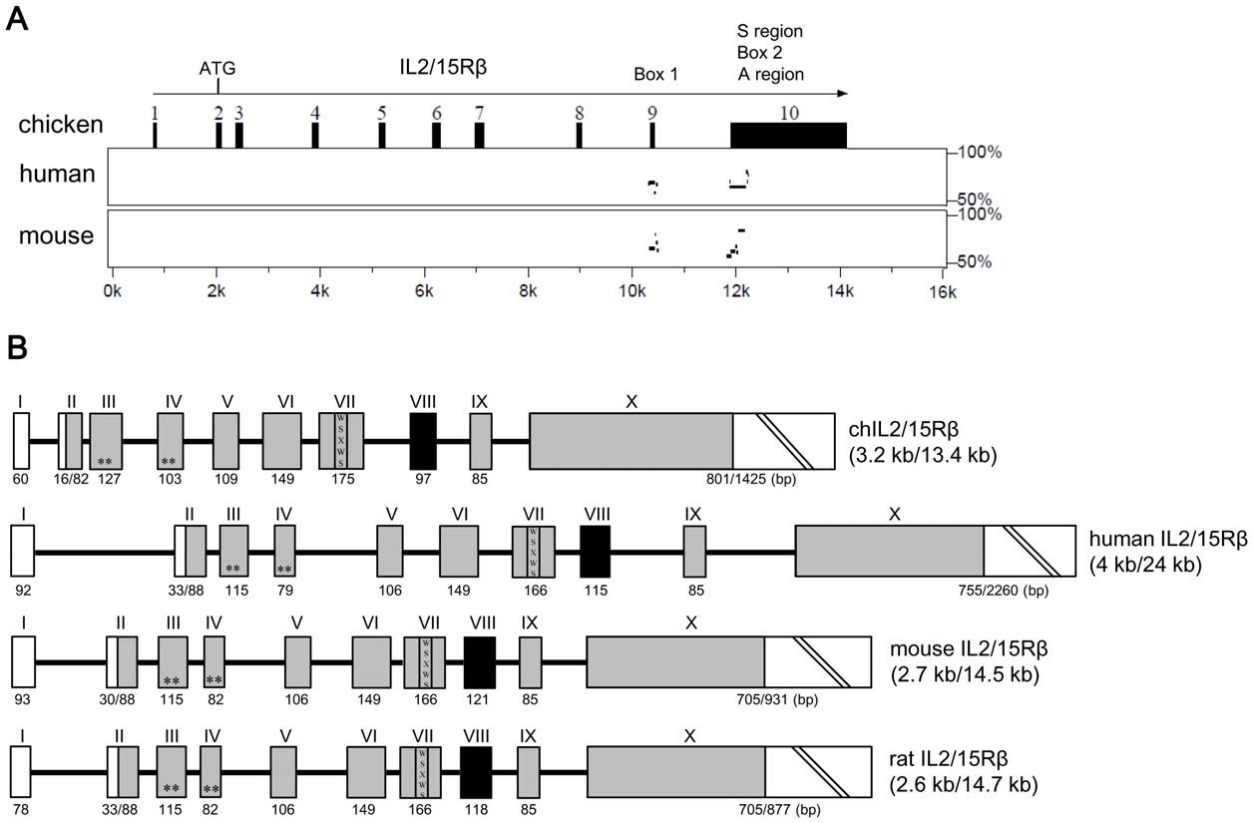
Percent identity plots and schematic comparison of the genomic regions of chicken and mammalian IL2/15Rβ genes. (A) Percent identity plots of the genomic regions of chicken and mammalian IL2/15Rβ genes using the PipMaker program. Regions bearing between 50% and 100% homology (*y*-axis) to human and mouse sequences are illustrated as dashes along 16 kb (*x*-axis) of the chIL2/15Rβ genomic sequences. Exons are shown by numbered black boxes. Direction of transcription is indicated by the arrow. ATG, transcription start site; S region, serine-rich region; A region, acidic region; Box 1, Box 1 domain; Box 2, Box 2 domain. (B) Schematic comparison of the genomic structures of chicken and mammalian IL2/15Rβ genes using Spidey program. Exons (boxes) are numbered at the top in Roman numerals. The numbers indicate lengths in base pairs encoded by each exon. White boxes, untranslated regions; grey shaded boxes, translated regions; black shaded boxes, putative transmembrane region; WSXWS, WSXWS motif. * conserved cysteine residues. The GenBank accession numbers used in this comparison were NM_000878 (human), NM_008368 (mouse), NM_013195 (rat) and JN642526 (chicken).

As shown in Figure 1A and 1B, chIL2/15Rβ contained four conserved cysteine residues and a WSXWS motif, both hallmarks of the type-I cytokine receptor superfamily [30]. The translated chIL2/15Rβ sequence contained five potential N-linked glycosylation sites (Asn-X-Ser/Thr) in the extracellular region. The large cytoplasmic region harbored signaling molecule binding sites, including PX(I/V)PXP(E/K) (Box 1) and (V/L)E(V/L)L (Box 2) motifs that are common to most hematopoietic cytokine receptors, and serine-rich (S region) and acidic-rich (A region) regions [30]–[32]. The S region (amino acids 301–354) contained nine serine residues. The A region (amino acids 347–416) contained 10 negatively-charged amino acids and one positively-charged amino acid. Amino acids 286–294 and 330–340 included Box 1 (LKIHIPDPE) and Box 2 (ISMLEVMQKND), respectively. Interestingly, the highest level of homology is located in the amino acid sequence of the cytoplasmic region with approximately 50% identity and 55–61% similarity (Fig. 1C). By percent identity plots of the genomic regions of chicken and mammalian IL2/15Rβ genes using the PipMaker program (http://pipmaker.bx.psu.edu/pipmaker/), regions bearing between 50% and 100% homology to human and mouse sequences are located in exons 9 and 10 including Box 1, Box 2, S and A regions (Fig. 2A).

**Table 1:** Amino acid identities (%, top right) and similarities (%, bottom left) of IL2/15Rβ chains from chickens, mammals and fishes.

	1	2	3	4	5	6	7	8
1. Chicken^1^		28.92	27.61	28.26	18.13	22.87	22.05	22.22
2. Human	38.23		60.29	59.96	22.22	25.16	24.83	25
3. Rat	35.45	67.81		83.33	23.36	26.79	25.81	26.96
4. Mouse	35.45	67.32	87.09		23.36	27.12	26.63	27.12
5. Zebrafish	27.28	32.51	33	34.15		38.88	38.23	39.7
6. Rainbow trout IL2Rβ1	32.02	35.94	36.27	36.6	49.01		85.13	92.64
7. Rainbow trout IL2Rβ2	30.88	35.62	35.29	36.11	47.87	87.41		83.82
8. Salmon	31.86	36.27	36.92	37.25	49.18	93.95	86.11	

1 The Genbank accession numbers used in comparison were JN642526 (chicken), NM_000878 (human), NM_013195 (rat), NM_008368 (mouse), NM_001128267 (zebrafish), FN813346 (rainbow trout IL2Rβ1), FN813347 (rainbow trout IL2Rβ1) and NM_001140548 (salmon).

### Genomic Structure of the chIL2/15Rβ Gene

The genomic clone spanned approximately 13.4 kb and consisted of ten exons and nine introns (Fig. 2B). The exon/intron organization of the chIL2/15Rβ gene was remarkably similar to its mammalian counterparts [33], [34], and all exon/intron boundaries contained consensus splice donor and acceptor sites. The extracellular domain was coded within exons 2–8, the transmembrane domain within exon 8, and the intracellular domain within exons 8–10. The four consensus cysteine residues were located in exons 3 and 4, two in each exon, and the WSXWS motif resided in exon 7. Interestingly, when compared with the genomic structures of the human, rat and mouse IL2/15Rβ genes, exons 6 and 9 were identical in length in all four sequences.

### Distribution of chIL2/15Rβ mRNA in Normal Tissues and Cell Lines

**Figure 3:**
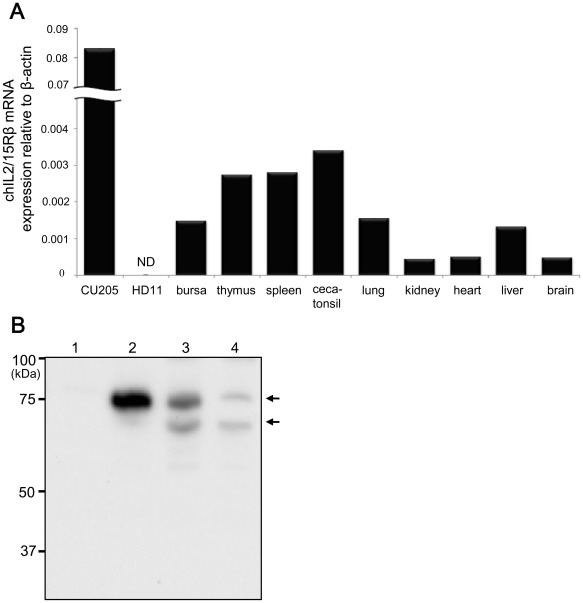
Distribution and molecular weight of chicken IL2/15Rβ. (A) Expression of chIL2/15Rβ transcripts in various chicken tissues and cell lines. Total RNA was isolated from various tissues of 10-day-old chickens and analyzed with quantitative real-time PCR. Tissue samples were pooled from five chickens. Expression levels were normalized to those of β-actin from the same samples. Data represent means of triplicate samples. Data are representative of two independent experiments with similar pattern results. CU205, REV-transformed lymphoblast cell line; HD11, macrophage cell line; ND, not detected. (B) Detection of chicken IL2/15Rβ protein with Western blot analysis. Whole-cell lysates of COS-7 cells were collected 48 h (lanes 1 and 2) after transient transfection with a chIL2/15Rβ-HA construct (lane 2) or empty pcDNA 3.1 (lane 1). To determine the size of the chIL2/15Rβ backbone, transfected cells were incubated for 24 h and then treated with 5 µg/ml tunicamycin as an inhibitor of N-linked glycosylation followed by incubation for an additional 6 h (lane 3) and 24 h (lane 4). Cell lysates from COS-7 cells were separated by SDS-PAGE under reducing conditions. Arrows indicate specific bands. Data are representative of three independent experiments with similar pattern results.

Quantitative real-time PCR analysis was used to examine the expression of chIL2/15Rβ transcripts in various normal tissues and two chicken cell lines, the REV-transformed lymphoblast cell line CU205 and the macrophage cell line HD11 (Fig. 3A). The expression levels of chIL2/15Rβ transcripts were relatively high in thymus, spleen and ceca-tonsil. Moderate expression was also identified in bursa, lung and liver with low expression levels identified in kidney, heart and brain. Of the two cell lines, only CU205 expressed a high level of chIL2/15Rβ transcripts.

### Molecular Weight of chIL2/15Rβ

Chicken IL2/15Rβ gene included eight N-glycosylation sites including five N-glycosylation sites in the extracellular region compared to four N-glycosylation sites in mammalian IL2/15Rβ genes such as human, mouse and rat. Thus, molecular weights of chIL2/15Rβ were identified in tunicamycin-treated COS-7 cells transfected with a chIL2/15Rβ-HA construct. As shown in Figure 3B, molecular weights of chIL2/15Rβ were similar to those observed in mammals. The 62.5 kDa protein (lower arrow) is likely the backbone of chIL2/15Rβ, whereas the 75 kDa protein (upper arrow) represents an N-linked glycosylated form of the protein.

### Quantitative Analysis of chIL2/15Rβ mRNA Expression in ConA-activated Splenic Lymphocytes and Tissues from *E. tenella*-infected Chickens

**Figure 4:**
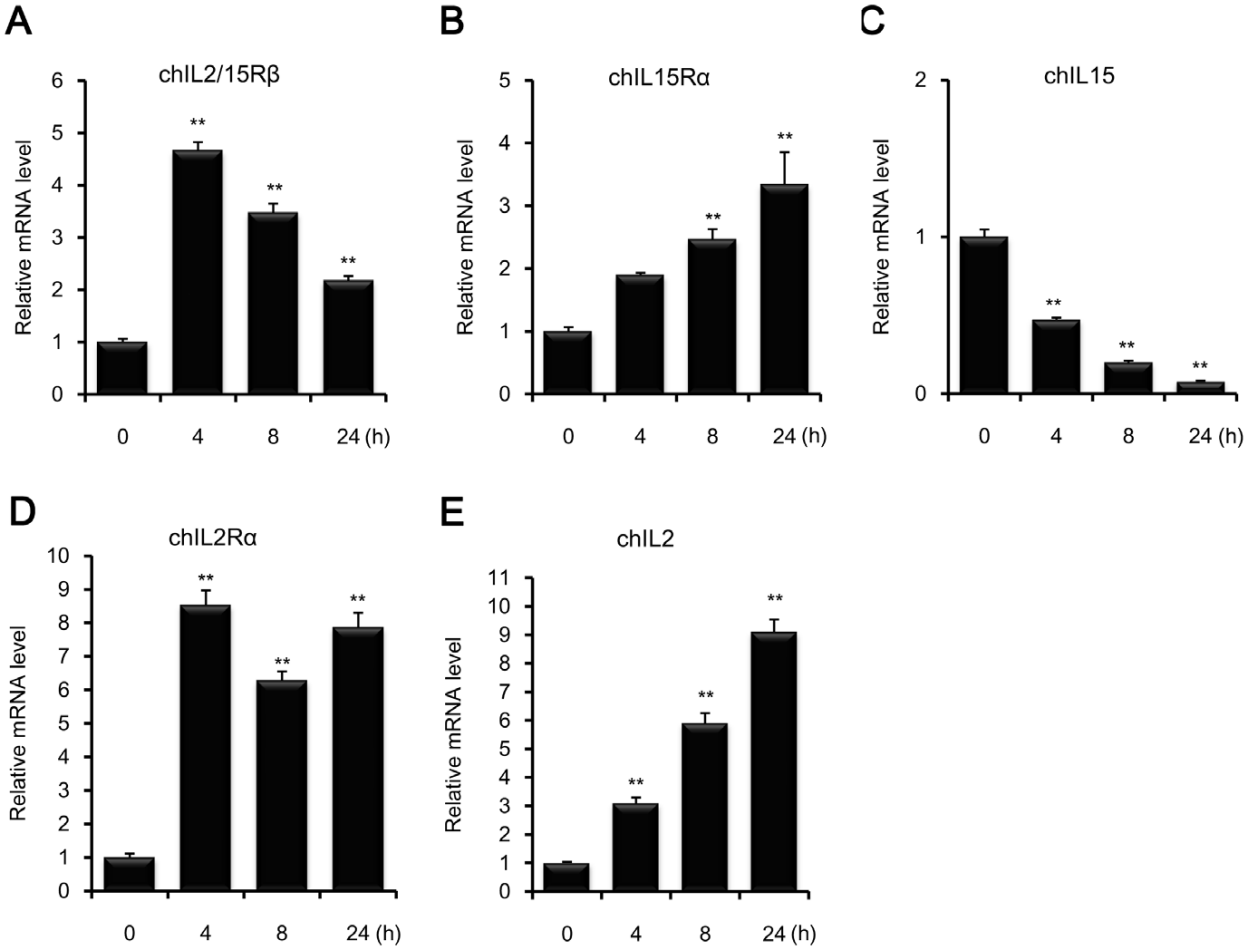
mRNA expression profiles of chIL2/15Rβ and related cytokines and receptors in ConA-stimulated splenic lymphocytes. Splenic lymphocytes were isolated from 2-week-old chickens, activated with 10 µg/ml ConA for the indicated times, and analyzed by quantitative real-time PCR. Expression levels were normalized to those of β-actin from the same samples. The *y* axis represents the fold change in expression of each gene from activated lymphocytes as compared to normal lymphocytes. Data represent means ± standard error of triplicate samples. Data are representative of three independent experiments with similar pattern results. ** *P*<0.01 was considered significant compared to untreated lymphocytes.

**Figure 5:**
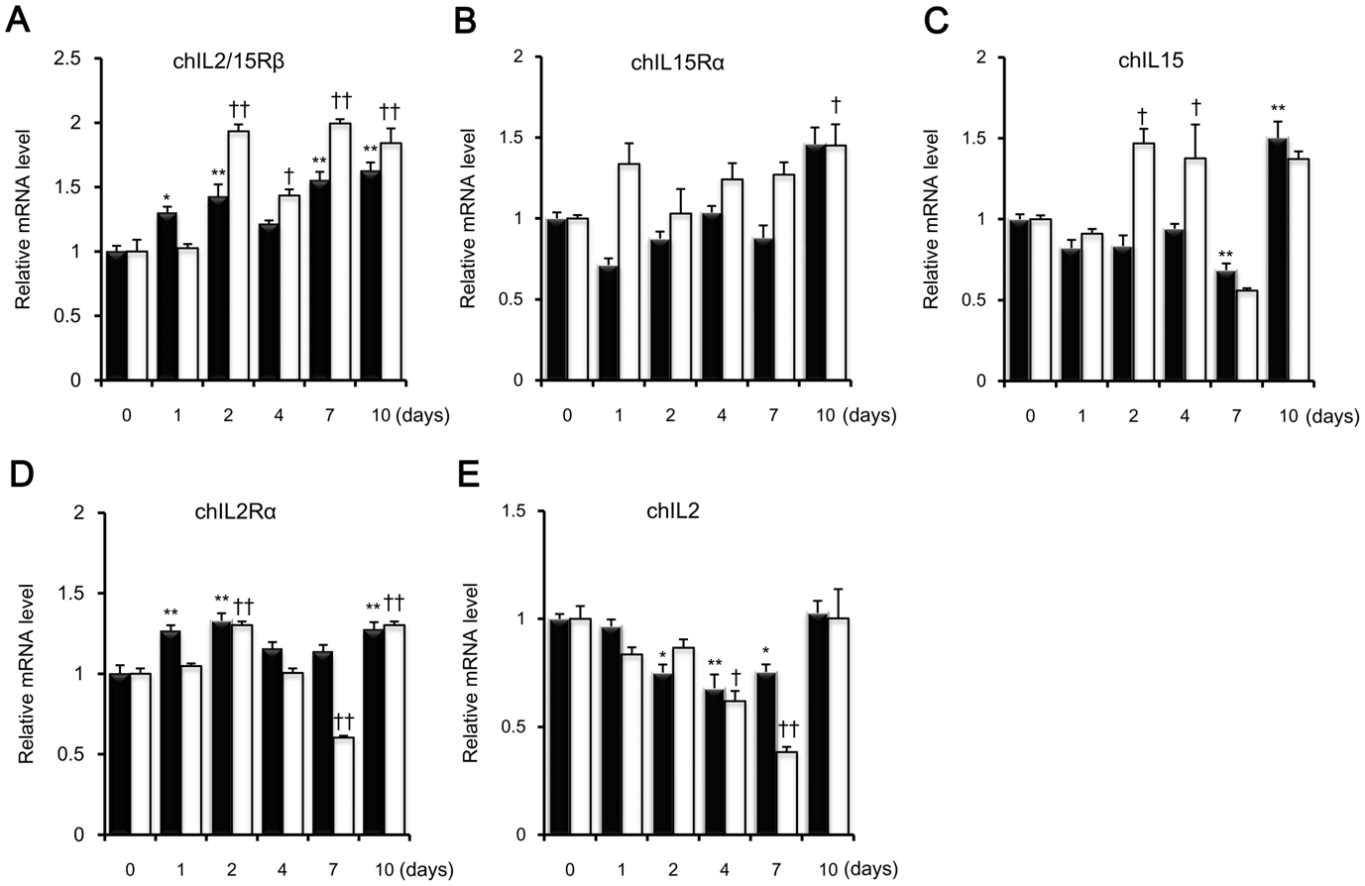
mRNA expression profiles of chIL2/15Rβ and related cytokines and receptors in *E. Tenella*-infected chickens. Ten-day-old chickens were orally infected with 1×10^4^ sporulated *E. tenella* oocysts. Spleens (closed bar) and ceca-tonsils (open bar) were collected on days 0, 1, 2, 4, 7, and 10. Tissue samples were pooled from five chickens and subjected to quantitative real-time PCR. Expression levels were normalized to those of β-actin from the same samples. The *y* axis represents the fold change in expression of each gene from *E. tenella*-infected chickens as compared to uninfected chickens. Data represent means ± standard error of triplicate samples. Data are representative of two independent experiments with similar pattern results. * *P*<0.05 or ** *P*<0.01 when compared to spleens of uninfected chickens. † *P*<0.05 or †† *P*<0.01 when compared to ceca-tonsils of uninfected chickens.

Using quantitative real-time PCR, expression profiles of chIL2/15Rβ, IL2, IL15, IL2Rα, and IL15Rα were determined in ConA-stimulated splenic lymphocytes (Fig. 4) and in the ceca-tonsils and spleens of chickens infected with *E. tenella* (Fig. 5). As shown in Figure 4, expression levels of the three receptors, chIL2/15Rβ, chIL2Rα, and chIL15Rα, were always elevated in ConA-activated splenic lymphocytes compared to normal splenic lymphocytes. The expression level of chIL2/15Rβ peaked after 4 h of ConA stimulation and then gradually decreased (Fig. 4A). Expression levels of chIL2Rα were higher than those of chIL15Rα (Fig. 4B, D). Interestingly, chIL2 and chIL15 mRNA showed inverse expression patterns in splenic lymphocytes following ConA activation; chIL2 mRNA expression increased while chIL15 mRNA levels decreased in time-dependent manners. Compared with control splenic lymphocytes, chIL2 and chIL15 transcript levels 24 h after ConA activation were 9.1 and 0.08 times higher, respectively (Fig. 4C, E), showing an approximately 114-fold difference in expression levels between the two cytokines.

In *E. tenella*-infected chickens (Fig. 5), expression levels of chIL2/15Rβ and chIL2Rα transcripts were, in general, up-regulated or unchanged in both ceca-tonsils and spleens compared to those of healthy controls. However, chIL2Rα mRNA expression was down-regulated in ceca-tonsils on day 7 (Fig. 5A, D). Expression of chIL15Rα and chIL15 transcripts was increased in ceca-tonsils, but not in spleens, where chIL15Rα and chIL15 mRNA levels decreased slightly except on day 10 after infection (Fig. 5B, C). chIL2 mRNA expression was remarkably reduced in ceca-tonsils and spleens until day 7, after which chIL2 mRNA levels returned to control levels by day 10 post-infection (Fig. 5E). Collectively, these results suggest that IL15 and its receptors play an imortant function in *Eimeria* infections.

**Figure 6:**
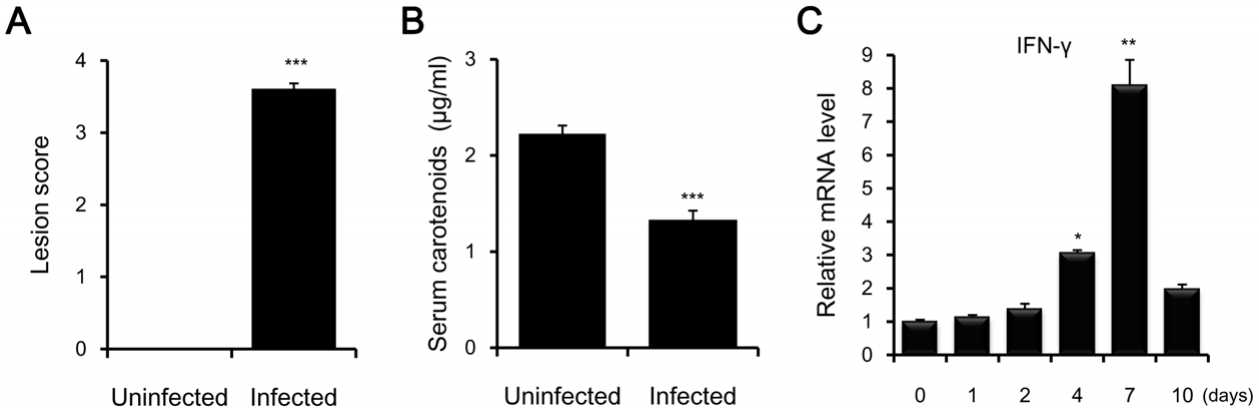
Determination of gut lesion scores, serum carotenoid levels, and IFN-γ transcript levels. Ten-day-old chickens were orally infected with 1×10^4^ sporulated *E. tenella* oocysts. Nine chickens were randomly chosen for serum samples and gut lesion scoring 7 days after *Eimeria* infection. (A) Lesion scores (0–4) were based on scoring techniques previously described (Johnson and Reid, 1970). (B) Serum samples were extracted with ten volumes of acetone to precipitate proteins. Absorbencies of the supernatants were determined spectrophotometrically at 456 nm using a β-carotene standard. Bars represent the means ± standard error from nine chickens. (C) Expression of IFN-γ mRNA in cecal-tonsils of chickens infected with *E. tenella*. Tissue samples were pooled from five chickens and subjected to quantitative real-time PCR. Expression levels were normalized to those of β-actin from the same samples. The *y* axis represents the fold change in expression of IFN-γ gene from *E. tenella*-infected chickens as compared to uninfected chickens. Data represent means ± standard error of triplicate samples. * *P*<0.05, ** *P*<0.01 or *** *P*<0.001 was considered significant compared to uninfected chickens. Data are representative of two independent experiments with similar pattern results.

On day 7, expression levels of chIL15 mRNAs, but not chIL15Rα, were dramatically reduced in ceca-tonsils of *E. tenella*-infected chickens (Fig. 5B, C). Thus, intestinal lesion scores, serum carotenoid levels, and interferon (IFN)-γ transcript levels were additionally monitored post-infection. As shown in Figure 6A, intestinal lesion scores increased significantly (*P*<0.001) in *E. tenella*-infected chickens as compared to uninfected chickens. Serum carotenoid levels were reduced significantly in infected chickens (*P*<0.001) (Fig. 6B). IFN-γ transcript expression was approximately 8.1-fold higher in infected chickens on day 7 as compared to control chickens (Fig. 6C). Given that the infections with *E. tenella* were successful and that the samples were prepared properly, the reduction of chIL15 mRNA may be caused by significant apoptosis of damaged intraepithelial cells (IECs), although additional studies would be needed to determine if the changes seen here are due to changes in transcription or transcript stability.

## Discussion

IL2/15Rβ functions as the signal-transducing component of the IL2/15R complex. Here, a full-length cDNA encoding chIL2/15Rβ was cloned. When compared to mammalian sequences, chIL2/15Rβ showed higher conservation levels than did chIL2Rα and chγ_c_. When compared to their mammalian counterparts, chIL2Rα and chγ_c_ had different molecular characteristics, especially with regard to exon numbers and alternative splicing [22], [24]. In contrast, the exon/intron structure was very similar between mammalian and chicken IL2/15Rβs [33], [34]. The number of conserved features and domains of the cytoplasmic region of IL2/15Rβ between different species suggest that chIL2/15Rβ plays an important role in IL2- and IL15-mediated signaling events. First, the large cytoplasmic region (300 amino acids) of chIL2/15Rβ, as compared to those of chIL2Rα (4 amino acids) and chγ_c_ (113 amino acids), provide binding sites for signaling molecules. Second, chIL2/15Rβ possesses the hallmark characteristics of mammalian IL2/15Rβ, such as the S and A regions and the Box 1 and Box 2 domains, whose locations were highly conserved. Cytoplasmic S and A regions are responsible for IL2-induced mitotic signaling and the physical association of p56^lck^, a src-family protein tyrosine kinase, respectively [35], [36]. The Box 1 and Box 2 domains play a crucial role in the association with Jak1 [32]. Using Multiple alignment and PipMaker analysis, comparison of nucleotide sequences indicated that Box 1, Box 2, S and A regions of chIL2/15Rβ gene shared with 60–75% identity to their mammalian counterparts, suggesting that signaling pathways engaged by IL2/15Rβ are similar between chickens and mammals. Third, a high degree of synteny was found between chicken and mammalian genomes, in which MPST, KCTD17, TMPRSS6, IL2/15Rβ, and C1QTNF6 were present in the same order on chicken chromosome 1, human chromosome 22, and mouse chromosome 15 (data not shown).

Expression of chIL2/15Rβ transcripts was high in lymphoid organs, including spleen, ceca-tonsil, and thymus. Interestingly, the CU205 cell line that expresses chicken T-cell receptor 3 (TCR3) and NK cell marker 28–4 antigen constitutively expressed chIL2/15Rβ [37], [38]. In the bursa, the site of B-cell development in chickens, only moderate levels of chIL2/15Rβ transcripts were detected. No chIL2/15Rβ transcripts were detected in the HD11 macrophage cell line with quantitative real-time PCR analysis suggesting a minimal interaction of IL2 with chIL2/15Rβ as seen in mammals [39], [40]. Prior studies have shown that although murine primary macrophages and cell lines express IL2/15Rβ in their resting states, IL2 concentrations that are necessary for biological activity are at the nanomolar level (K_d_ >10 nM), indicating an absence of high-affinity IL2R complexes in macrophages [39], [40]. Because chIL2/15Rβ transcripts were mainly expressed in lymphoid organs and cell lines composed of NK and T cells, not B cells and macrophages, tissue-specific expression patterns of IL2/15Rβ are similar between chickens and mammals [6], [7].

Human IL2/15Rβ has a calculated molecular size of 58.3 kDa and an actual translated size of 65–77 kDa that increases to 85–92 kDa upon cross-linking with IL2 [41], [8]. N-linked glycosylation and sialylation of human IL2/15Rβ are well established and supported in that endoglycosidase F and neuraminidase treatments diminish the molecular weight of human IL2/15Rβ from 70–82 kDa to a broader range of 60–78 kDa [42]. Our data suggest post-translational modification of chIL2/15Rβ due to the calculated molecular size of 61.4 kDa. As shown in Figure 3B, a shift in chIL2/15Rβ size was observed after treatment with tunicamycin, an inhibitor of glycosylation.

chIL2 induces T-cell proliferative activity, and its transcripts are expressed in ConA-activated spleen T cells [20]. ConA activation of T cells also induces cell-surface expression of chIL2R [43]. The interaction between IL2 and its receptors stimulates the growth, differentiation, and survival of antigen-selected cytotoxic T cells by autocrine or paracrine methods [1]. Considering the critical role of IL2/15Rβ in IL2-mediated signal transduction and the formation of high- and intermediate-affinity IL2R complexes, increased chIL2/15Rβ transcript levels may be necessary to ensure a continued supply of chIL2/15Rβ for the formation of higher affinity receptor complexes during mitogenic activation. In contrast, chIL15 is down-regulated in spleen cells activated with ConA [21]. In our data, chIL2 and chIL15 expression levels were inversely related as chIL2 expression increased and chIL15 expression decreased following ConA activation of splenic lymphocytes. These findings suggest that chIL2/15Rβ signaling was driven by chIL2 rather than chIL15 in ConA-activated splenic lymphocytes. Interestingly, the expression kinetics of the components of the chIL2/15R complex were quite different. The expression of chIL2/15Rβ increased 4.6 times after 4 h of mitogen stimulation with a subsequent decline, whereas transcript levels of chIL2Rα, chIL15Rα, and chIL2 steadily increased indicating independent gene regulation of chIL2/15Rβ.

The mRNA expression profiles of the chIL2/15R complex were examined in ceca-tonsils and spleens following oral challenge with live *E. tenella* oocysts. Because *E. tenella* mainly infects the cecum, local immunity mediated by cecal tonsil lymphocytes is important in coccidiosis [44], [45]. Our data demonstrated the possibility that lymphocytes in ceca-tonsils were more responsive to IL15 than spleen lymphocytes from *E. tenella*-infected chickens. In ceca-tonsils, overall mRNA levels of chIL2/15Rβ, chIL15Rα, and chIL15 were elevated, suggesting that the interactions of these molecules in local hosts play an important role in immune responses to intracellular parasites.

Interestingly, chIL15 expression levels were significantly down-regulated 7 days post-infection with *E. tenella,* whereas chIL2/15Rβ and chIL15Rα levels steadily rose. Intraepithelial cells (IECs) constitutively express IL15 mRNA and protein [46]. IL15 synthesis in IECs is up-regulated in *L. monocytogenes*-infected rats [47], and intestinal γδ T cells express high levels of IL15Rα mRNA and proliferate in response to IL15 in mice [48]. In addition, IL2/15Rβ expression is induced on CD8αβ^+^NK1.1^+^ T cells in the lamina propria (LP) of transgenic mice; IL15 is preferentially expression in small IECs [49]. During *E. tenella* infection, IECs represent the first line of defense and undergo severe damage, especially 6–8 days after infection [50]. The reduction of chIL15 seven days post-infection may be caused by significant apoptosis of damaged IECs rather than intestinal intraepithelial lymphocytes (IELs) or LP lymphocytes.

The intestinal damage induced by *E. tenella* infection was accompanied by changes in the expression of chIL2Rα or chIL2. These observations can be explained by the immunophysiological regulation of IL2 production that can prevent autoimmunity [1]. Indeed, when chickens are given an external source of chIL15, but not chIL2, levels of CD3^+^ T cells are elevated and accompanied by reduced oocyst shedding and body weight loss [51], [52]. Taken together, the growth and activation of IELs or LP lymphocytes in response to signaling through chIL2/15Rβ is preferentially regulated by chIL15 rather than chIL2 during *E. tenella* infection.

Here, we cloned chIL2/15Rβ and examined its expression patterns and functions. Molecular analysis indicated that chIL2/15Rβ possesses a number of conserved features and cytoplasmic domains as compared to mammalian IL2/15Rβ. Furthermore, chIL2/15Rβ transcripts are mainly expressed by NK cells and T-cell subsets, but not on B cells and macrophages. Transcription of chIL2/15Rβ was elevated in the cecum during *E. tenella* infection, indicating an important role of IL15-mediated immunoregulation in local defenses against intracellular parasitism. The availability of recombinant chIL2/15Rβ and chIL2/15Rβ-specific antibodies for detection will enhance future study of the role of the IL2/15R complex in chickens and its evolutionary relationship among its mammalian counterparts.

## Materials and Methods

### Animals and Infections

Male cobb 500 chickens (Harim, Korea) were given unlimited access to feed and water. Constant light was provided for the duration of the experiments. Ten-day-old chickens were orally infected with 1×10^4^ sporulated *E. tenella* oocysts (Korean isolate 291–7) and transferred to disposable cages [53]. *E. tenella* were cleaned by flotation on 5.25% sodium hypochlorite and washed three times with PBS. All animal experiments protocols were approved by the Institutional Animal Care and Use Committee (IACUC) at Gyeongsang National University, Jinju, Republic of Korea (Approval Number: GNU-LA-34).

### Cloning of chIL2/15Rβ cDNA

Chicken spleens were dilacerated with a syringe plunger through a cell strainer (SPL Life Sciences, Korea) to obtain single-cell suspensions in Hank’s balanced salt solution (HBSS) (Sigma-Aldrich, USA). Total RNA was extracted from splenic lymphocytes using RiboEx reagent (Geneall, Korea) and treated with RNase-free DNase I (Fermentas, Canada). Single-stranded cDNA was synthesized from total RNA with oligo dT primers using a Transcriptor First Strand cDNA Synthesis Kit (Roche Applied Science, Germany).

Based on a chIL2/15Rβ EST sequence (http://www.ensembl.org/; accession number: ENSGALG00000012472), 5′/3′-Rapid Amplification of cDNA Ends (RACE) was performed with chIL2/15Rβ-specific primers (for 5′ RACE, 5′-TCTTCTGCATCACCTCCAGC-3′; for 3′ RACE, 5′-CCTCCCCATTCTCCACATCT-3′) with splenic lymphocyte cDNA using a 5′/3′ RACE kit (5′/3′ RACE 2^nd^ Generation; Roche Applied Science) according to the manufacturer’s protocol. PCR products were cloned into TA vectors (RBC, Taiwan) and sequenced (Macrogen, Korea). PCR was performed on a DNA Engine thermocycler (Bio-Rad, USA) as follows: 5 min at 95°C, 30 cycles of 1 min at 95°C, 1 min at 55°C, and 2 min at 72°C, and a final 5 min extension at 72°C. The cDNA sequence was submitted to GenBank with accession number JN642526.

### Cell Culture

Chicken lymphoblast cell line CU205 [37], [54], macrophage cell line HD11 [37], [55], and splenic lymphocytes were cultured in Dulbecco’s modified eagle’s medium (DMEM) (Hyclone, USA) supplemented with 10% FBS and penicillin/streptomycin (10,000 unit/ml) (Hyclone) at 41°C in 5% CO_2_. Splenic lymphocytes were resuspended to 5 × 10^6^ cells/ml and stimulated with 10 µg/ml ConA (Amersham Bioscience, Sweden).

### Expression of chIL2/15Rβ in COS-7 Cells

Full-length chIL2/15Rβ cDNA was amplified by PCR from single-stranded cDNA from splenic lymphocytes using the following primers: 5′-GATCAAGCTTCCAGAACAGATGAAGCCCTCCT-3′ and 5′-GATCTCTAGACTAAGCGTAATCTGGAACATCGTATGGGTAGACAGAGCCATGGCTGTATTG-3′ containing *Hin*d III and *Xba* I restriction enzyme sites (single underline) and the influenza virus hemagglutinin (HA) sequence (double underline). PCR products were digested with *Hin*d III and *Xba* I and cloned into the corresponding restriction sites of pcDNA3.1 (Invitrogen, USA). COS-7 cells [24] were transiently transfected with 10 µg constructs using Lipofectamine Reagent (Invitrogen) and incubated for 5 h in serum-free DMEM at 37°C in 5% CO_2_. FBS was then added to the growth medium to a final concentration of 10%. To determine the size of the chIL2/15Rβ backbone, transfected cells were incubated for 24 h and then treated with 5 µg/ml tunicamycin (Sigma–Aldrich) as an inhibitor of N-linked glycosylation followed by incubation for an additional 6 h and 24 h.

### Western Blot Analysis

Protein samples were mixed with equal volumes of sample buffer (0.125 M Tris-HCl, pH 6.8, 4% SDS, 20% glycerol, 10% 2-mercaptoethanol, and 0.004% bromophenol blue), heated for 4 min at 94°C, resolved on 10% SDS-polyacrylamide gels, and electroblotted onto polyvinyl difluoride (PVDF) membranes (Bio-Rad). Membranes were blocked with PBS containing 1% nonfat dry milk for 16 h at 4°C, incubated with monoclonal anti-HA antibody (Sigma–Aldrich) for 1 h, washed three times with PBS containing 0.05% Tween 20 (PBS-T), and incubated with horseradish peroxidase-conjugated goat anti-mouse IgG antibody (Promega, USA) in PBS containing 1% nonfat dry milk for 40 min at room temperature. Membranes were washed five times with PBS-T followed by five washes with distilled water, visualized using an enhanced chemiluminescence (ECL) kit and Western Blotting Detection Reagents (GE Healthcare Life Sciences) and exposed to X-ray film.

### Quantitative Real-time PCR

Normal tissues, cell lines, ConA-activated splenic lymphocytes and tissue samples pooled from five chickens infected with *E. tenella* were subjected to real-time PCR analysis in triplicate. cDNA synthesis was performed using random hexamer primers. Real-time PCR was performed on a CFX96 real-time PCR system (Bio-Rad) with SYBR Green (Bioneer, Korea) using the primers listed in Table 2. A melting curve was obtained at the end of each run to identify that there was a single amplification product and no primer dimers. Standard curves were generated using serial, 5-fold dilutions of ConA-activated splenic lymphocyte cDNA. The relative expression levels of individual transcripts were normalized to those of β-actin with Bio-Rad CFX software. The gene expression levels were quantified using the comparative ΔCt or ΔΔCt method with β-actin gene as a reference for normalization. The fold change in expression of each gene examined from *E. tenella*-infected chickens was calculated relative to their expression levels in the same tissues of uninfected chickens.

**Table 2:** List of primers used in quantitative real-time PCR.

RNA target	Primer and sequence	Efficiency	References
IL2/15Rβ	(For) 5'-TTCTTTCCTCCACTCATCTCT-3'	1.952	JN642526
	(Rev) 5'-GCATCACCTCCAGCATTG-3'		
IL15Rα	(For) 5'-AGTTTCCACCCAGACCTT-3'	1.843	XM_414982
	(Rev) 5'-GTCACTGCCACCACATAG-3'		
IL2Rα	(For) 5'-TGGACACCCGTTGATAGG-3'	1.912	NM_204596
	(Rev) 5'-GCAGAATGGAGAAGCAGAAT-3'		
IL15	(For) 5'-TGTTCTTCTGTTCTGAGTGATG-3'	1.821	NM_204571
	(Rev) 5'-GTTGGTACTGGAGACAAATACTT-3'		
IL2	(For) 5'-TTCTGGGACCACTGTATGCTCTT-3'	-	[58]
	(Rev) 5'-TACCGACAAAGTGAGAATCAATCAG-3'		
IFN-γ	(For) 5'-GCCGCACATCAAACACATATCT-3'	-	[59]
	(Rev) 5'-TGAGACTGGCTCCTTTTCCTT-3'		
β-actin	(For) 5'-CACAGATCATGTTTGAGACCTT-3'	-	[60]
	(Rev) 5'-CATCACAATACCAGTGGTACG-3'		

### Determination of Gut Lesion Scores and Serum Carotenoid Levels

Nine chickens were randomly selected for gut lesion scoring and serum analysis 7 days after *Eimeria* infection. Lesion scores were based on scoring techniques previously described [56]. Each chicken received a numerical value from 0 to 4. Lesion scores were evaluated by three independent observers. To precipitate proteins from serum samples, sera were extracted with ten volumes of acetone. Samples were vortexed, centrifuged for 10 min at 2,800×*g*, and stored at 4°C for 1 h in the dark. Supernatant absorbencies were determined spectrophotometrically at 456 nm using a β-carotene standard as previously described [57].

### Sequence Analysis

The predicted signal peptide sequence and transmembrane region were identified using the SignalIP program (www.cbs.dtu.dk/services/SignalP) and TMpred program (www.ch.embnet.org/software/TMPRED_form.html), respectively. Theoretical pI value and predicted molecular weight were calculated using Compute pI/Mw (http://web.expasy.org/compute_pi/). Amino acids multiple alignment was determined using ClustalW2 program (www.ebi.ac.uk/Tools/msa/clustalw2) and shaded using GeneDoc program (www.nrbsc.org/gfx/genedoc/) and identity and similarity were calculated using SIAS program (http://imed.med.ucm.es/Tools/sias.html). mRNA to genomic sequence alignment was performed using Spidey program (www.ncbi.nlm.nih.gov/spidey/). Percent identity plot (PIP) analysis of the genes between multiple mammalian and avian species was carried out using MultiPipMaker (http://pipmaker.bx.psu.edu/pipmaker/) [61].

### Statistical Analysis

Data were calculated by Student’s *t*-test or one-way ANOVA followed by Dunnet multiple comparison test using InStat® software (Graphpad, San Diego, CA). Differences were considered significant at *P*<0.05. The data were expressed as mean value ± standard error.
